# Prolongation of Fate of Bacteriophages In Vivo by Polylactic-Co-Glycolic-Acid/Alginate-Composite Encapsulation

**DOI:** 10.3390/antibiotics11091264

**Published:** 2022-09-17

**Authors:** Sang-Guen Kim, Sib Sankar Giri, Su-Jin Jo, Jeong-Woo Kang, Sung-Bin Lee, Won-Joon Jung, Young-Min Lee, Hee-Jin Kim, Ji-Hyung Kim, Se-Chang Park

**Affiliations:** 1 Laboratory of Aquatic Biomedicine, Research Institute for Veterinary Science, College of Veterinary Medicine, Seoul National University, Seoul 08826, Korea; 2 Department of Food Science and Biotechnology, Gachon University, Seongnam 13120, Korea

**Keywords:** PLGA, alginate hydrogel, encapsulation, bacteriophage, long-circulation, fate, pharmacokinetics, prophylaxis, phage therapy

## Abstract

With concern growing over antibiotics resistance, the use of bacteriophages to combat resistant bacteria has been suggested as an alternative strategy with which to enable the selective control of targeted pathogens. One major challenge that restrains the therapeutic application of bacteriophages as antibacterial agents is their short lifespan, which limits their antibacterial effect in vivo. Here, we developed a polylactic-co-glycolic acid (PLGA)/alginate-composite microsphere for increasing the lifespan of bacteriophages in vivo. The alginate matrix in PLGA microspheres encapsulated the bacteriophages and protected them against destabilization by an organic solvent. Encapsulated bacteriophages were detected in the tissue for 28 days post-administration, while the bacteriophages administered without advanced encapsulation survived in vivo for only 3–5 days. The bacteriophages with extended fate showed prophylaxis against the bacterial pathogens for 28 days post-administration. This enhanced prophylaxis is presumed to have originated from the diminished immune response against these encapsulated bacteriophages because of their controlled release. Collectively, composite encapsulation has prophylactic potential against bacterial pathogens that threaten food safety and public health.

## 1. Introduction

The global mortality rate has reached 700,000, and it is predicted to increase to 10 million by 2050 [1,2]. This rapid increase in deaths is thought to originate from the prevalence of multidrug-resistant (MDR) bacterial pathogens. With their bacteriolytic potential, lytic bacteriophages have been suggested as potential therapeutic candidates [3]. The lysogenic characteristics that alter the physiological features of host bacteria (i.e., mobility and pathogenicity) have recently been of interest in the attempt to control bacteria [4]. For these reasons, the use of bacteriophages has been proposed as a promising strategy against MDR bacterial infections; a number of commercial bacteriophage products are available and clinical trials have begun [5,6,7,8].

However, therapeutic strategies involving bacteriophages have a major limitation; bacteriophages are naturally cleared by the human immune system or environmental stress factors before they can find a susceptible bacterial host to infect in order to replicate [9]. To circumvent this problem and achieve optimal efficacy, the repetitive administration of bacteriophages has been recommended [10,11,12]. Furthermore, regardless of the potency of bacteriophages, repetitive exposure to non-self-antigens can elicit a boosted immune response [13]. Thus, maintaining “long-circulating” bacteriophages that conserve their infectivity and increasing their lifespan are tactics that minimize the number of bacteriophage doses required for the effective application of bacteriophages as therapeutic agents [14].

The enabling of bacteriophages to endure environmental stresses can be accomplished through encapsulation [15]; for example, alginate encapsulation has been widely applied for bacteriophage encapsulation owing to their ease of production and biocompatibility [16,17,18]. The delivery of bacteriophages through alginate encapsulation with their “egg-box” structure could provide protection against gastric fluids. Another approach is the slowly hydrolyzing polymer, polylactic-co-glycolic acid (PLGA), which has significant potential for sustained release [19]. Several previous reports described the application of PLGA for food-safety and therapeutic purposes as they provide long-term protection with the help of encapsulating materials [20,21]. However, alginate and PLGA also have limitations; the former cannot protect the encapsulated material under harsh conditions (i.e., high acidity) and the latter has a detrimental effect on bacteriophages’ infectivity owing to the organic solvent(s) used in the production process [15].

Here, we describe a composite hydrogel matrix that allows the slow release of bacteriophages in vivo to prolong their lifespan. This composite hydrogel matrix makes use of biodegradable plastic PLGA and a naturally derived polymer, alginate, to overcome the vulnerability of bacteriophages to the organic solvents used in the process. We determined the efficacy of the sustained release of bacteriophages encapsulated in PLGA/alginate-composite microspheres. We found that our bacteriophage encapsulation strategy can prevent early clearing; it protected the model animal against the target pathogen for 28 days after a single administration.

## 2. Materials and Methods

### 2.1. Microbial Culture Condition

The bacterial strain (*Aeromonas hydrophila* JUNAH) and its bacteriophage (pAh-6C) were isolated in our laboratory previously [22,23]. *Aeromonas hydrophila* was cultured on tryptic soy agar (Difco) or tryptic soy broth (Difco). For bacteriophage culture, tryptic soy broth containing 0.4% (*w*/*v*) agar was used as the top agar and assayed with overnight-grown host (JUNAH) strain. The microorganisms were incubated for 18–24 h at 25 °C.

### 2.2. Propagation and Purification of Bacteriophage

The bacteriophages were propagated and purified following the protocol described by Kim et al. [24]. Briefly, host bacteria (2 × 10^8^ colony-forming unit [CFU]/mL) and the bacteriophage (2 × 10^5^ plaque-forming unit [PFU]/mL) were co-cultured for 18 h for the propagation. Next, the bacterial debris were excluded after centrifugation (12,000× *g*, 10 min), crude bacteriophage lysate was precipitated with polyethylene glycol 8000 (sigma)/NaCl (sigma, ≥99%), and the resuspended pellet was purified using a CsCl (sigma, ≥98%) gradient. The bluish-white bacteriophage band was collected and dialyzed using a 7000 MWCO dialysis bag. The purified bacteriophage solution (>10^10^ PFU/mL) was stored at 4 °C until further use.

### 2.3. Preparation of Microsphere

Water-in-oil-in-water (W/O/W) double-emulsion microspheres were prepared according to a previously described protocol [25] with some modifications. To prepare the PLGA microspheres, 500 μL of the bacteriophage solution (3 × 10^9^ PFU) in phosphate buffered saline (PBS; 137 mM NaCl, 2.7 mM KCl, 8 mM Na_2_HPO_4_, and 2 mM KH_2_PO_4_) was mixed (at 4000× *g* for 1 min) with 210 mg PLGA (sigma, lactide:glycolide 75:25, mol wt 66,000–107,000) dissolved in 3 mL dichloromethane (sigma, ≥99.8%) to form a primary W/O emulsion. The primary emulsion was poured into a 50-milliliter polyvinyl alcohol (PVA; sigma, ≥99%, mol wt 89,000–98,000) solution (4% *w*/*v*) and homogenized at 3000 rpm for 2 min to form a secondary W/O/W emulsion. For the PLGA/alginate-composite microspheres, the bacteriophage solution was mixed with a sodium-alginate solution to prepare a 1% alginate-bacteriophage solution (3 × 10^9^ PFU). This solution was mixed (3200 rpm for 1 min) with PLGA (210 mg) dissolved in dichloromethane (3 mL) to form a primary W/O emulsion. The primary emulsion was poured into a 50-milliliter PVA solution (4% *w*/*v*) and homogenized at 3000 rpm for 1 min to form a secondary W/O/W emulsion. Next, the calculated volume of 0.2 mL calcium chloride solution (500 mM) was added to the secondary W/O/W emulsion to crosslink the alginate, and the mixture was homogenized for 1 min. Deionized water (50 mL) was slowly added to the secondary emulsion over the course of 30 min. To facilitate the evaporation of the organic solvent (dichloromethane), the emulsion was further stirred at 300 rpm for 8 h. Microspheres were obtained after evaporation. They were washed twice with PBS, harvested by centrifugation at 5000× *g* for 10 min, and re-suspended in PBS for further use.

### 2.4. Bacteriophage-Encapsulation Rate of Microsphere

To determine the encapsulation capacity of the PLGA or PLGA/alginate-composite microspheres, PFUs in the release plateau (P) were deducted from the initially loaded PFUs (I). The encapsulation efficiency was calculated using the following formula:

$$\mathrm{Encapsulation}\;\mathrm{efficiency}\;(\%)=\frac{I-P}{I}\times 100$$

### 2.5. Scanning Electron Microscopy of Microsphere

The morphology of the microspheres was examined using a field-emission scanning electron microscope (FE-SEM; ZEISS, Oberkochen Germany) at 15 kV. The microspheres were mounted on a stub and coated with platinum for observation. Their size distribution and hydrodynamic diameter were measured by laser diffraction using an LS 13 320 particle-size analyzer (Beckman Coulter, Brea, CA, USA).

### 2.6. In Vitro Release Assay

To determine the kinetics of bacteriophage release from the microspheres, 100 mg bacteriophage-encapsulated microspheres were resuspended and incubated in 10 mL PBS at 25 °C and 150 rpm. The microspheres were initially harvested from the PBS solution at 0.5, 1, 3, 6, and 12 h. Thereafter, the microspheres were harvested at 1, 2, 3, 5, 7, 15, 21, 28, 40, 50, and 60 days. For each harvest, the solution was replaced with the same volume of PBS. After the harvest, the resulting solution was assayed by the double-layer method to determine cumulative release of bacteriophages. A solution of native bacteriophages (2 × 10^8^ PFU/mL) was assayed as a positive control at the same time intervals and under similar experimental conditions. The experiment was performed with triple replicates (*n* = 3).

### 2.7. Animal Experiments

Animal experiments were approved by the Institutional Animal Care and Use Committee (IACUC) of Seoul National University (SNU-200109-3). Healthy cyprinid loaches with mean body weight were purchased from a commercial fish farm in Gyeonggi province, South Korea, and acclimated in the aquatic animal facility of the Laboratory of Aquatic Biomedicine, College of Veterinary Medicine, Seoul National University. Before the experiment, fish were kept in a 200-liter tank at 25 ± 1 °C, with a light schedule of 12 h, and fed with commercial feed at 2% body weight daily for 20 days. For the experiment, 5 or 10 fish were allocated per group in a 2-liter aquarium (20 cm × 45 cm).

### 2.8. Sample Collection

Before collecting samples from the animals, either anesthesia was induced or euthanasia was performed with tricaine mesylate, as described previously [26]. Blood was collected from the caudal vein, and the spleen was collected from the aseptically opened peritoneal cavity. For the enumeration of PFUs, the blood samples were assayed by the double-layer method before clotting, and the concentration unit was expressed as PFU per milliliter of blood. Spleen samples were weighed and ground in 1 mL PBS for the double-layer assay. The concentration unit for the spleen sample was PFU per gram tissue. For the serum-agglutination assay, serum was harvested from the blood samples by centrifugation at 6500× *g* for 10 min at 4 °C. The serum samples were stored at −20 °C until further use.

### 2.9. Bacteriophage Administration and In Vivo Fate Assay

As PLGA/alginate-composite microspheres are superior to PLGA microspheres in vitro, only the composite microspheres were examined in vivo, with native bacteriophages as the controls. To observe the fate of bacteriophages in vivo, fish were administered with native bacteriophages or PLGA/alginate-encapsulated bacteriophages at a concentration of 2 × 10^8^ PFU/fish through intraperitoneal injection. At 6 h and 1, 2, 3, 5, 7, 15, 21, 28, 40, 50, and 60 days post-administration, blood and spleen samples were collected after euthanizing the fish, as described above (*n* = 5).

### 2.10. Challenge Assay

To determine the prophylactic effect of the administered bacteriophages or PLGA/alginate composite, fish were injected with LD_50_ or LD_100_ (dosage required to kill 50 or 100% of fish) of JUNAH at 1, 3, 7, 15, 28, 40, 50, and 60 days post-administration (*n* = 10), as previously described [22]. The mortality rate was monitored for 7 consecutive days.

### 2.11. Serum-Agglutination Assay

To examine the immunogenicity of the native and encapsulated bacteriophages, fish were intraperitoneally inoculated with unencapsulated or PLGA/alginate-encapsulated bacteriophages at a concentration of 2 × 10^8^ PFU/fish. To induce secondary humoral immunity, a second inoculation of bacteriophages was performed 4 weeks post-primary inoculation. Blood samples were collected 2 and 4 weeks post-inoculation (first and second inoculation groups), and 8 weeks post-inoculation (first inoculation group only). Serum samples were prepared as described above, and the agglutination assay was performed in a round-bottom 96-well microplate, as previously described, with minor modifications [27]. Briefly, samples were serially diluted 2-fold in sodium magnesium buffer (100 mM NaCl, 50 mM Tris pH 7.5, and 10 mM MgSO4), and the same volume of purified bacteriophages (2 × 10^8^ PFU/mL) was added to each well. The microplates were incubated overnight and observed for agglutination. The lowest concentration of antibody that caused agglutination was reported as the titer value.

### 2.12. Statistical Analysis

Statistical significance was analyzed using one-way analysis of variance (ANOVA) with Tukey post hoc test in SigmaPlot v.12.0 (Systat Software, Inc., Chicago, IL, USA). A probability value below 0.05 was considered significant.

## 3. Results and Discussion

### 3.1. Encapsulation and Release In Vitro

A total of 5 × 10^9^ PFU of pAh6-C was encapsulated with the polymers, resulting in approximately 100 mg of microspheres. The fate assay was performed using microspheres suspended in PBS solution for 60 days. Owing to its very slow hydrolyzing feature, PLGA is frequently used for encapsulating materials for extended release (ER) [19]. However, the encapsulation potential of the PLGA microspheres was low (0.06%), as an organic solvent should be used for producing the microsphere (Table 1) [28]. The detrimental effect of organic solvents on bacteriophages has been proposed as a limiting factor for the PLGA encapsulation of bacteriophages, despite its ER characteristic [15,29]. Thus, water-soluble biopolymers that do not deteriorate bacteriophage infectivity are generally proposed for bacteriophage encapsulation [17,30]. However, these swell easily and cause the rapid release of the bacteriophages retained in the matrix (i.e., within minutes or hours). Consequently, they cannot protect the bacteriophages [29]. This results in short-term protection from environmental factors without the ER effect, as the native bacteriophages can also circulate through the body for a few days.

**Figure 1:**
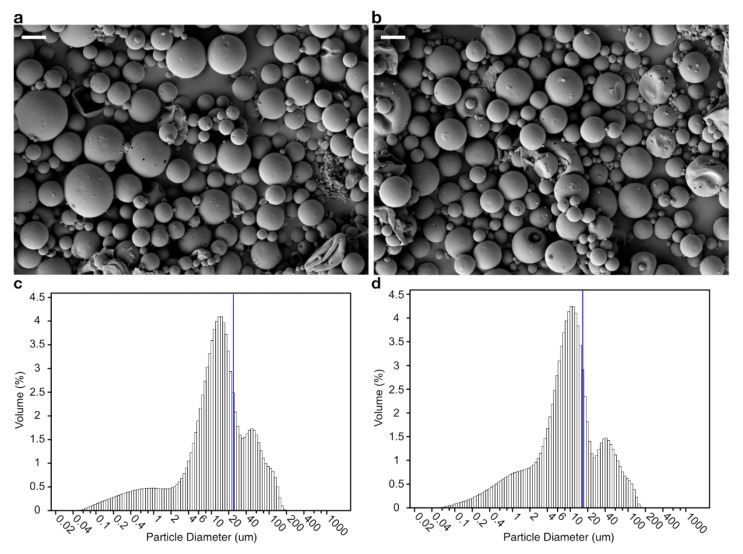
Morphological characteristics of the microsphere. Scanning electron microscope (SEM) images of the bacteriophage-loaded polylactic-co-glycolic acid (PLGA) microsphere (**a**), and PLGA/alginate-composite microsphere (**b**). Scale bar, 10 μm. Size distribution of the bacteriophage-loaded PLGA microsphere (**c**), and PLGA/alginate-composite microsphere (**d**). The blue line represents mean size of the microspheres.

To minimize the direct contact between the bacteriophages and the organic solvent at the water–organic solvent interface, we combined the bacteriophages with alginate, a gelatinous polymer; the encapsulation efficiency of the composite microspheres was considerably higher (71.74%) compared with the PLGA encapsulation. Morphologically, the microsphere shapes did not differ between the PLGA and the PLGA/alginate-composite microspheres (Figure 1a, b); however, the average size of the PLGA microspheres was 34% larger than that of the composite microspheres (Table 1, Figure 1c,d).

**Figure 2:**
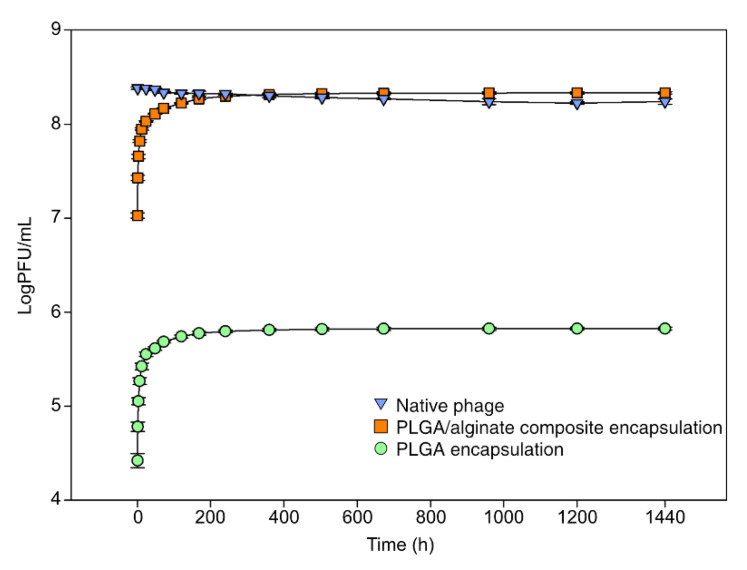
Cumulative release profiles of encapsulated bacteriophages in vitro (mean ± SEM, *n* = 3). Blue, orange, and green represent native-, polylactic-co-glycolic acid (PLGA)/alginate-composite-encapsulated, and PLGA-encapsulated bacteriophages, respectively.

Generally, it is believed that larger microspheres can accommodate more and secrete at a slower rate compared with smaller microspheres as they have larger surface areas. We found that although their size was small, more bacteriophages could be encapsulated in the PLGA/alginate-composite microspheres with a slower release rate. An initial burst was observed for both encapsulations within 10 days (240 h), releasing 92–93% of the encapsulated bacteriophages (Figure 2; PLGA: 6 × 10^9^ PFU/mL, PLGA/alginate: 2 × 10^9^ PFU/mL).

### 3.2. Fate of Bacteriophage In Vivo

**Figure 3:**
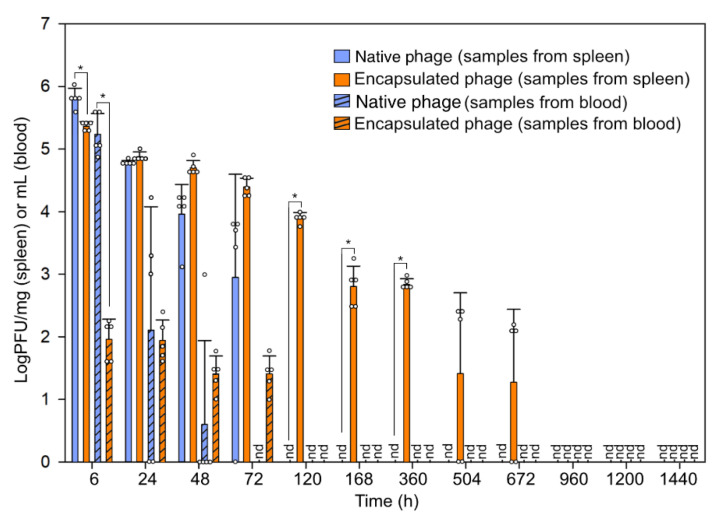
Fate of bacteriophages in vivo (mean ± SEM, *n* = 5). nd: not detected. Blue and orange represent native and polylactic-co-glycolic acid (PLGA)/alginate-encapsulated bacteriophages, respectively. Bars without and with stripes represent samples from spleen and blood, respectively. Asterisk (*) indicates statistical significance.

To determine the pharmacokinetics of pAh6-C, infective bacteriophages were collected from the blood and spleen for 60 days. While the infectivity of pAh6-C was intact for a long time in vitro, its in vivo fate was shorter; bacteriophages were detected in the blood and spleen samples for 2 and 3 days, respectively (Figure 3; 2 × 10^2^ PFU/mL blood, 6 × 10^3^ PFU/g spleen), which was in accordance with previous studies [31,32]. Sometimes, the extended fate of bacteriophages in vivo can be accomplished by the “serial-passage technique”, which upregulates the circulating capacity by 10,000–20,000 times [14]. However, this strategy of repeated administration cannot be performed with bacteriophages that induce hyper-immune reactions (e.g., phiX174) [33]. We developed encapsulation that can be easily adapted to bacteriophages in general. As the PLGA/alginate-composite microspheres were superior to the PLGA microspheres in terms of encapsulation capacity, the former was applied in the in vivo assay to determine the lifespans of the bacteriophages. An extended fate of the bacteriophages was observed in vivo, which revealed much slower release than in vitro (Figure 3). Interestingly, infective bacteriophages were detected in the spleen 28 days after the administration of composite-encapsulated bacteriophages (8 × 10 PFU/g spleen). The extended fate of the bacteriophages in vivo is considered to have been due to the combined effect of the prolonged release of the bacteriophages from the slowly rupturing matrix and the matrix protecting the phages from contact with neutralizing antibodies [34,35,36].

### 3.3. Prophylaxis of Encapsulated Bacteriophages

The prophylactic use of antibiotics in food animals remains widespread, despite the negative consequences [37]. Thus, the prophylactic use of bacteriophages to replace antibiotics has been eagerly discussed since the prophylactic effect was confirmed [38]. As administered bacteriophages are eliminated within days in vivo, practical applications are not feasible until bacteriophages’ fate in vivo is extended. Furthermore, a previous report indicated that immediate treatment with native pAh6-C after injecting the pathogen could prevent host mortality (100% survival rate) against a LD_50_ dose of pathogen, and lowered the mortality by 16% against a LD_100_ dosage [22]. Although short-lived, the prophylactic effect of the bacteriophage pAh6-C was observed for a few days (3 to 7 days; Table 2). Furthermore, encapsulation with PLGA/alginate-composite matrix prolonged the fate of the bacteriophages, which protected the animals against the pathogenic bacterial challenge (Table 2). The administration of the encapsulated pAh6-C 28 days before infection ameliorated mortality in both the LD_50_- and LD_100_- injected groups (Table 2). Circulating bacteriophages below the detection limit of the quantitative method can reduce the mortality of challenged animals. Furthermore, as PLGA/alginate-composite encapsulation extended the fate of the pAh6-C from 3 to 28 days in vivo, prophylaxis could also be extended to 28 days.

### 3.4. Humoral Immune Reaction against the Bacteriophage Administration

**Figure 4:**
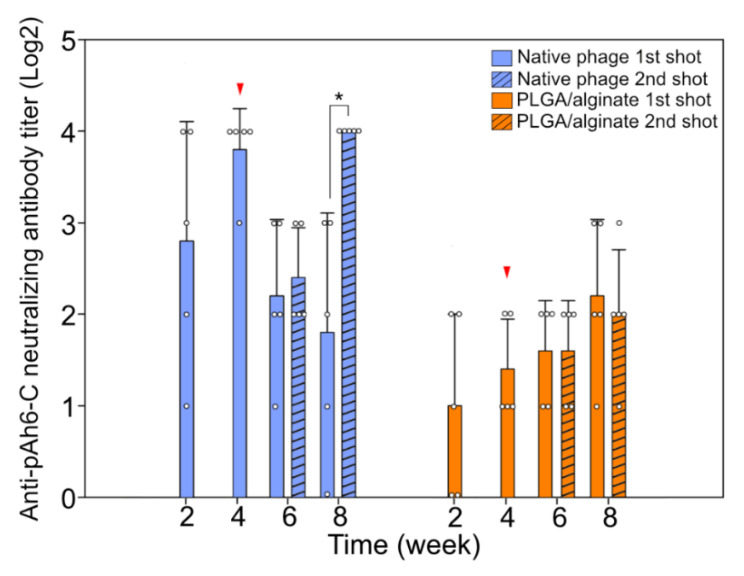
Antibody-mediated humoral immunity against bacteriophage administration (mean ± SEM, *n* = 5). Second administration was performed 4 weeks after the first administration (indicated by the red arrow). Blue and orange bars represent native and polylactic-co-glycolic acid (PLGA)/alginate-encapsulated bacteriophage administered groups, respectively. Bars without and with stripes represent single (first)- or boost (second)-administered groups, respectively. Asterisk (*) indicates statistical significance.

No natural neutralizing antibody against the native bacteriophage pAh6-C was observed in the animals. Although the bacteriophage pAh6-C is not a strong immunogen, humoral immunity peaked at 4 weeks post-administration. The second administration boosted the generation of the neutralizing antibody for 4 additional weeks (Figure 4). This was consistent with previous studies, in which the repeated administration of bacteriophages induced faster and stronger anti-bacteriophage response(s) than single administration [39]. By contrast, the humoral immune reaction was considerably delayed for the encapsulated bacteriophages owing to their slow release. Encapsulation has been considered a powerful strategy for viral vectors to circumvent the immune responses of hosts [40]. Even with the slight effect of sustained release, the formation of the specific antibody could be hindered [41], and the immunogenic strength of the composite-encapsulated bacteriophages was significantly lower than that of bacteriophages without encapsulation. It is presumed that a low quantity of immunogens was exposed to the lymphocyte at a particular time [12]. Furthermore, the second administration of encapsulated bacteriophages did not induce a secondary humoral response against the bacteriophages. Whether encapsulation can protect bacteriophages from the entire immune system of the mammalian host remains unclear; however, the fact that encapsulated bacteriophages were more stable, as they were not neutralized by antibodies, cannot be ignored. Furthermore, the advantages of prolonged release (Figure 3) and decreased immunogenicity (Figure 4) owing to PLGA/alginate-composite encapsulation may make them more suitable for therapeutic applications.

## 4. Conclusions

Encapsulation matrices for bacteriophages have gained in interest owing to their protective effects against environmental factors (i.e., host immune system, gastric fluids). We developed a new strategy to advance bacteriophage encapsulation using the PLGA polymer combined with alginate hydrogel. The PLGA/alginate-composite matrix encapsulated a considerably higher concentration of bacteriophages than the matrix made solely of PLGA, which has been considered a limiting factor of encapsulation using the PLGA polymer. Moreover, the release rate of the former matrix was slower than that of the latter, although the dimension of the former was smaller than that of the latter. The release of infective bacteriophages encapsulated with the PLGA/alginate-composite matrix in vitro and in vivo was observed for extended periods (60 and 28 days, respectively). The attenuated antigenicity of the PLGA/alginate-composite-encapsulated bacteriophages enabled repeat administration. Thus, the encapsulation of the PLGA/alginate-composite matrix has significant potential for application in prophylaxis against bacterial pathogens. However, further work is required to determine the versatility of the PLGA/alginate-composite encapsulation of bacteriophages in other animals, including humans.

## Figures and Tables

**Table 1:** Physical characteristics of bacteriophage-loaded microsphere. Values are the means ± standard error of triple replicates.

Microsphere	Inner Aqueous Phase		Microsphere Size (μm)	Bacteriophage-Encapsulation Efficiency (%)
	PVA (%)	Alginate (%)		
PLGA	4	0	23.1566 ± 0.1855	0.2230 ± 0.0066
PLGA/alginate	0	1	17.1533 ± 0.0449 *	71.7444 ± 1.6024 *

Asterisk (*) indicates statistical significance.

**Table 2:** The extended prophylactic effect of bacteriophages with or without encapsulation (*n* = 10). Challenge with LD_50_ (a), and LD_100_ (b). The survival rate was observed for 7 days. ^a^ ND: not done.

(a) LD_50_ Challenge									
Survivability (%)		days post-administration							
		1	3	7	15	28	40	50	60
1st trial	Bacteriophage	70	80	50	30	40	50	50	40
	PLGA/alginate	90	90	80	70	60	50	50	50
2nd trial	Bacteriophage	80	60	60	50	50	40	50	40
	PLGA/alginate	90	80	90	80	70	50	40	50
(b) LD_100_ challenge									
Survivability (%)		days post-administration							
		1	3	7	15	28	40	50	60
1st trial	Bacteriophage	80	70	10	10	10	10	ND ^a^	ND
	PLGA/alginate	90	80	80	60	40	0	ND	ND
2nd trial	Bacteriophage	80	10	0	0	10	0	ND	ND
	PLGA/alginate	90	90	70	70	30	0	ND	ND

## Data Availability

All data generated or analyzed during this study are included in this published article.
